# Supplementary material for: The Deubiquitinating Enzyme USP20 Regulates the TNFα-Induced NF-κB Signaling Pathway through Stabilization of p62
Source: Int J Mol Sci. 2020 Apr 28;21(9):3116. doi: 10.3390/ijms21093116 (PMC7247158; doi:10.3390/ijms21093116)
Supplement: Supplementary file 1 [file ijms-21-03116-s001.pdf]

Supplementary Materials

The deubiquitinating enzyme USP20 regulates TNFα-induced NF-κB signaling pathway through the stabilization of p62

Jihoon Ha, Minbeom Kim, Dongyeob Seo, Jin Seok Park, Jaewon Lee, Jinjoo Lee, Seok Hee Park

<Table of Contents>

1. Supplementary figures; Figure S1 – S3
2. Supplementary tables; Table S1 and S2

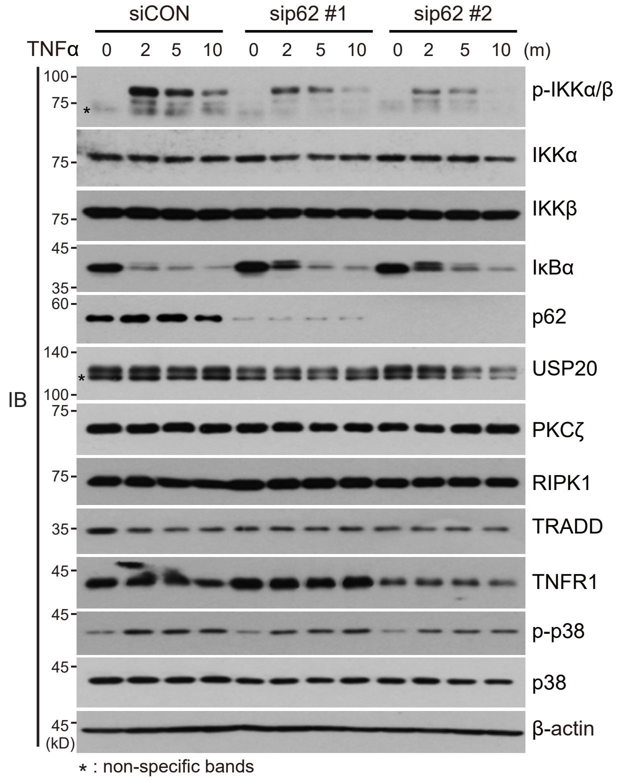

Figure S1. p62 depletion decreases TNFα-induced NF-κB signaling.
HeLa cells were reverse-transfected with 20 nM control siRNA (siCON) or two independent p62-specific siRNAs (sip62 #1 and sip62 #2). Cells were treated with 10 ng/ml TNFα for the indicated time points. Total cell lysates were immunoblotted with the indicated antibodies. Expression of β-actin was used as a loading control. The immunoblot image is representative of three independent experiments.

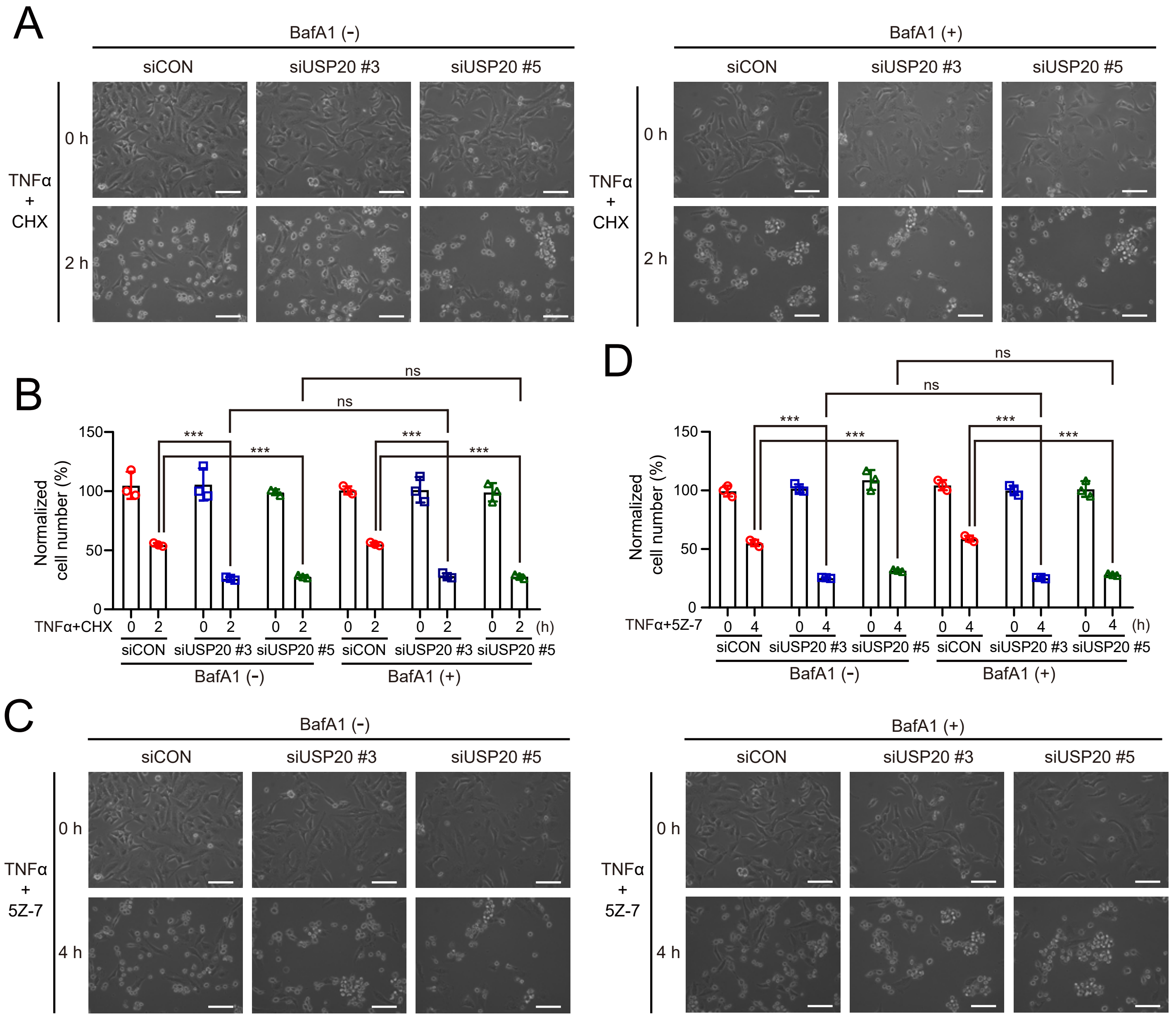

Figure S2. Autophagy is not related to TNFα-mediated apoptosis under USP20 depletion.
A, B: USP20-depleted and control (siCON) HeLa cells were pre-treated with 100 nM bafilomycin A1 (BafA1) and subsequently treated with 20 ng/ml TNFα plus 10 μg/ml cycloheximide (CHX) for 2 h. Morphological changes were observed by light microscopy (**A**). Scale bars, 1000 μm. Live cell countings (**B**) were performed to measure cell viability. **C, D:** USP20-depleted and control (siCON) HeLa cells were pre-treated with 100 nM bafilomycin A1 (BafA1) and subsequently treated with 20 ng/ml TNFα plus 1 μM 5Z-7 (TAK1 inhibitor) for 4 h. Morphological changes were observed by light microscopy (**C**). Scale bars, 1000 μm. Live cell countings (**D**) were performed to measure cell viability. The data in this figure were statistically analyzed by two-way ANOVA followed by Bonferroni's multiple comparison test (****P* < 0.001 compared to the control cells without TNFα/CHX or TNFα/5Z-7, ns; not significant, n = 3). The bars represent the mean ± SD. The images in (**A**) and (**C**) are representative of three independent experiments.

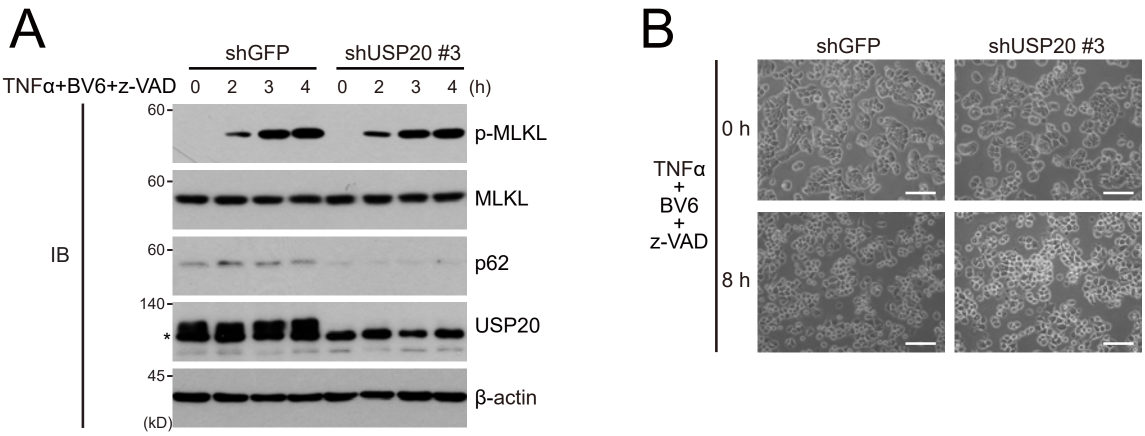

Figure S3. USP20 depletion is not related to necroptosis.
A, B: USP20-depleted HT29 cells were generated by infections of lentiviruses expressing specific shRNA against endogenous *USP20* mRNA. As a negative control, lentiviruses expressing shRNA against *GFP* mRNA (shGFP) were used. USP20-depleted and control (shGFP) HT29 cells were treated together with 30 ng/ml TNFα, 1 μM BV6 and 20 μM z-VAD-fmk for 8 h. Cell lysates were immunoblotted with the indicated antibodies (**A**). Morphological changes were observed by light microscopy (**B**). Scale bars, 1000 μm. The images in this figure are representative of three independent experiments.

Table S1. Primer sequences used for real-time RT-PCR in this study.

Gene	Species	Direction	Sequences
GAPDH	Human	Forward	5'-TGTAGTTGAGGTCAATGAAGGG-3'
		Reverse	5'-ACATCGCTCAGACACCATG-3'
USP20	Human	Forward	5'-CAATGGGCAGTGGTACGAGT-3'
		Reverse	5'-CCGCGAAGGTGTTGAACTTG-3'
p62	Human	Forward	5'-GTGGCTGTAACCTGCTGGAT-3'
		Reverse	5'-CTCTTTCAGGGACAGGCTGG-3'
BFL1	Human	Forward	5'-AGGTCCAAGCAAAACGTCCA-3'
		Reverse	5'-ATCCACATCCGGGGCAATTT-3'
cFLIP	Human	Forward	5'-CATAAGCCGTTTGACCACGC-3'
		Reverse	5'-TGAACCGCTTCACGCCTAAT-3'

Table S2. The sequences of siRNAs used in this study.

siRNA	Species	Sequences
sip62 #1	Human	Sense : 5'- CAUGUCCUACGUGAAGGAUGAUU-3'<br>Antisense : 5'-AAUCAUCCUUCACGUAGGACAUG-3'
sip62 #2	Human	Sense : 5'-GCAUUGAAGUUGAUAUCGAUUU-3'<br>Antisense : 5'-AAAUCGAUAUCAACUUCAAUGC-3'
siUSP20 #3	Human	Sense : 5'-GCGAGUGGCUCAACAAGUU-3'<br>Antisense : 5'-AACUUGUUGAGCCACUCGC-3'
siUSP20 #5	Human	Sense : 5'-GCCAGAACGUGAUCAAUGG-3'<br>Antisense : 5'-CCAUUGAUCACGUUCUGGC-3'
